# Pathway and Mechanism of pH Dependent Human Hemoglobin Tetramer-Dimer-Monomer Dissociations

**DOI:** 10.1371/journal.pone.0081708

**Published:** 2013-11-28

**Authors:** Yao-Xiong Huang, Zheng-Jie Wu, Bao-Tian Huang, Man Luo

**Affiliations:** Department of Biomedical Engineering, Ji Nan University, Guang Zhou, China; Illinois Institute of Technology, United States of America

## Abstract

Hemoglobin dissociation is of great interest in protein process and clinical medicine as well as in artificial blood research. However, the pathway and mechanisms of pH-dependent human Hb dissociation are not clear, whether Hb would really dissociate into monomers is still a question. Therefore, we have conducted a multi-technique investigation on the structure and function of human Hb *versus* pH. Here we demonstrate that tetramer hemoglobin can easily dissociate into dimer in abnormal pH and the tetramer → dimer dissociation is reversible if pH returns to normal physiological value. When the environmental pH becomes more acidic (<6.5) or alkaline (>8.0), Hb can further dissociate from dimer to monomer. The proportion of monomers increases while the fraction of dimers decreases as pH declines from 6.2 to 5.4. The dimer → monomer dissociation is accompanied with series changes of protein structure thus it is an irreversible process. The structural changes in the dissociated Hbs result in some loss of their functions. Both the Hb dimer and monomer cannot adequately carry and release oxygen to the tissues in circulation. These findings provide a comprehensive understanding on the pH-dependent protein transitions of human Hb, give guideline to explain complex protein processes and the means to control protein dissociation or re-association reaction. They are also of practical value in clinical medicine, blood preservation and blood substitute development.

## Introduction

In certain abnormal metabolism and respiration conditions, especially in some pathological situations such as acidosis or alkalosis, the pH value of human blood would fall outside of the normal physiological range of 7.35-7.45. Some preserved blood specimens in blood bank even have pH values lower than 6.5 owing to various factors[1,2]. It was reported that under abnormal pH condition, human tetramer hemoglobin would dissociate into dimers[3–9]. The dissociation of tetrameric hemoglobin is a very important issue in clinic and blood substitute research because dissociated Hb in body circulation would induce some side effects such as renal tubular damage and toxicity[10], and be cleared rapidly from circulation[11] . However, up till now, the mechanism of the dissociation is not completely clear. Whether the Hb dimer would further dissociate into monomers is also a question for lack of solid evidence, although several hypotheses have been made.

Only Shaeffer[12] and Mrabet[13] reported that they had found experimentally some free 3H-labeled α chain monomers were incorporated into carbonmonoxy-hemoglobin A dimers by exchanging with pre-existing unlabeled α chain subunits in a pH range of 6.5-8.4. They believed that it indicated dissociation of Hb dimers into monomers. Regardless of whether such an indirect evidence is convincing or not, the dimer–monomer dissociation constant K_2_ calculated from their experiment was just 4.7×10^-13^ M, suggesting that the possibility of the dissociation was very low in the circumstance. In other words, the dissociation was mainly a random behavior, and did not involve any specific factor which induced change in the hemoglobin structure thus causing the dissociation. 

To our knowledge, besides no direct evidence has been reported to indicate whether normal human hemoglobin (Hb A) would dissociate to monomer in solutions of abnormal pH, no one knows if dissociated hemoglobin can re-associate to a normal tetramer structure when pH returns to physiological level. Obviously, clarification of these questions is significant. Since it can not only lead to a comprehensive understanding on human hemoglobin dissociations, but also provide methods of controlling the protein dissociation or re-association reaction, and give guidelines to clinical medicine and blood preservation, as well as blood substitute development.

Therefore, we performed a multi-technique systematic study on the issue. Our objective was to obtain direct evidences on (1) the size distribution of Hb molecules in the solutions with various pH values; (2) the pathways of human hemoglobin dissociation; (3) the protein structural changes under different dissociation conditions; and (4) the structural changes of the protein’s globin and heme vs. pH. Thereby we can reveal the mechanism of various Hb dissociations, and disclose whether the dissociation processes are reversible when the solution pH returns to normal physiological value. 

## Materials and Methods

The protocol of this Study was approved by Ji Nan University Animal Care and Use Committee conforming to the Chinese Public Health Service Police on Human Care and Use of Laboratory Animals. Informed written consent was obtained from the healthy non-smoking adult volunteers providing normal blood.

### Reagents

All reagents and solutions were prepared from analytical grade materials. 20 mM phosphate buffer was prepared in distilled water; its pH was adjusted to the desired values ranging from pH 5.4 to 9.0 by adjusting different proportion of Na_2_HPO_4_ and NaH_2_PO_4_. 

### Preparation of hemoglobin

Hemoglobin was prepared according to Geraci et al[14]. Normal blood was obtained from healthy non-smoking adult volunteers (providing informed written consent) by venipuncture and poured into heparinized tubes. The blood was centrifuged at 1500 rpm for 10 min, during which the plasma and buffy coat were removed by aspiration. Thereafter the erythrocytes were washed three times with 0.9% NaCl, and then centrifuged for 10 min at 2000 rpm to remove the supernatant. The erythrocytes were later kept cold at 4 °C for 15min and lysed in 10 vol of ice-cold water, then churned to be disrupted. The sample was kept at 4 °C again for 20min, afterwards, the hemolysate was obtained by centrifugation for 30min at 12000rpm (4 °C), followed by filtration through a 0.22-μm disposable filter holder. The obtained samples were passed over a Sephadex G-25 for purification. Later, the purified hemoglobin was stored in a refrigerator at 4 °C and ready to be used.

The concentrations of hemoglobin in each of the sample solutions were determined using the hemiglobincyanide (HiCN) method and detected by an absorption spectrophotometry at 540nm[15]. The purified hemoglobin was diluted by the phosphate buffers of different pH values (5.4, 5.9, 6.2, 7.4, 8.0 and 9.0) to be 100 μM (heme) in concentration. The concentration was sufficiently low so that inter-particle interactions could be neglected[16].

### Dynamic light scattering

Considering none of the conventional techniques such as gel chromatography, gel filtration, sedimentation etc.[3–8] can perform non-disturbance, real time, *in situ* measurement on the natural situation about the dissociation of Hb in solutions, we improved the technique of dynamic light scattering (DLS)[17] to obtain detailed information about the size distribution of Hb molecules in solution. In the measurements, the scattered light intensity and its autocorrelation function were measured by a Zeta PALS instrument (Brookhaven, USA) using a laser with wavelength of λ= 678 nm. Detailed procedures of the DLS measurements were the same as that we described previously[18]. The diameters of the hemoglobin in different pH values were detected by analyzing the autocorrelation function of the scattered light. The size distribution of Hb molecules in each pH was derived from a NNLS(Non-Negatively constrained Least Squares algorithm) analysis using “number” weighting by which each particle (no mater it is small or large) has equal weighting once the final distribution is calculated. By this means, the proportion of each kind of Hb molecules (monomers, dimers and tetramers) in the solutions can be calculated accurately. All the reported size distribution data are the averages of the results from at least five measurements. The measurements were performed at temperature of 37 °C.

### Gel chromatography

To verify the information obtained by DLS, we employed gel chromatography to determine if the solutions really coexisted hemoglobin molecules of different molecular weights. Large zone elution volumes were determined as a function of pH for human hemoglobin. A Sephadex G-50 column (1.6×40cm) was employed with the eluent buffer: 20mM phosphate buffer (pH7.0). Cytochrome C (Sigma, MW=13KDa) and bovine serum albumin (Sigma, MW = 67KDa) were used as the references to determine the elution volumes of human hemoglobin in different solutions.

### UV-visible absorption spectrophotometry

We also performed UV-Visible absorption spectroscopy to measure the structure of heme and its combination with globin chain as functions of pH. The UV-visible absorption spectrophotometry was taken with a Unicam 500(Thermal, USA) in the wavelength ranging from 350 to 700 nm at room temperature. The heme peaks of hemoglobin as functions of pH values were obtained by analyzing the UV-visible spectra measured in different pH suspensions.

### Raman spectroscopy

To perform non-disturbance, real-time measurements on the molecular structure and oxygen carrying capacity of hemoglobin in various pH solutions, we also developed a series of novel methods of Raman confocal micro-spectroscopy[19,20]. The Raman spectra measurement was performed using a RAM INV (Horiba JY, France) system with an inverted Olympus optical microscope. The 514 nm light from an Ar^+^ iron laser was chosen as the excitation light for it has the advantage in obtaining resonant signal at the characteristic oxidation marker ν4 band (1358cm^-1^) of hemoglobin and providing direct and detailed information about the structure of heme[19–22]. The spectra were recorded between 1800 and 600 cm^-1^ with a resolution of 1 cm^-1^. For each measurement, a laser exposure time of 10 s was selected and ten scans were accumulated. Before the measurements, a silicon wafer with a character band at 520.7cm^-1^ was used to calibrate the instrument on daily basis. All the spectra were normalized using the CH_2_ band at 1448cm^-1^ as normalization metric. The measurements were performed at 37 °C. For every pH value, five parallel samples were prepared and each sample was measured at least five times, so the spectrum of each pH value was obtained from the average result of at least 25 spectra.

## Results and Discussion

### Sizes of hemoglobin under different pH values

**Figure 1:**
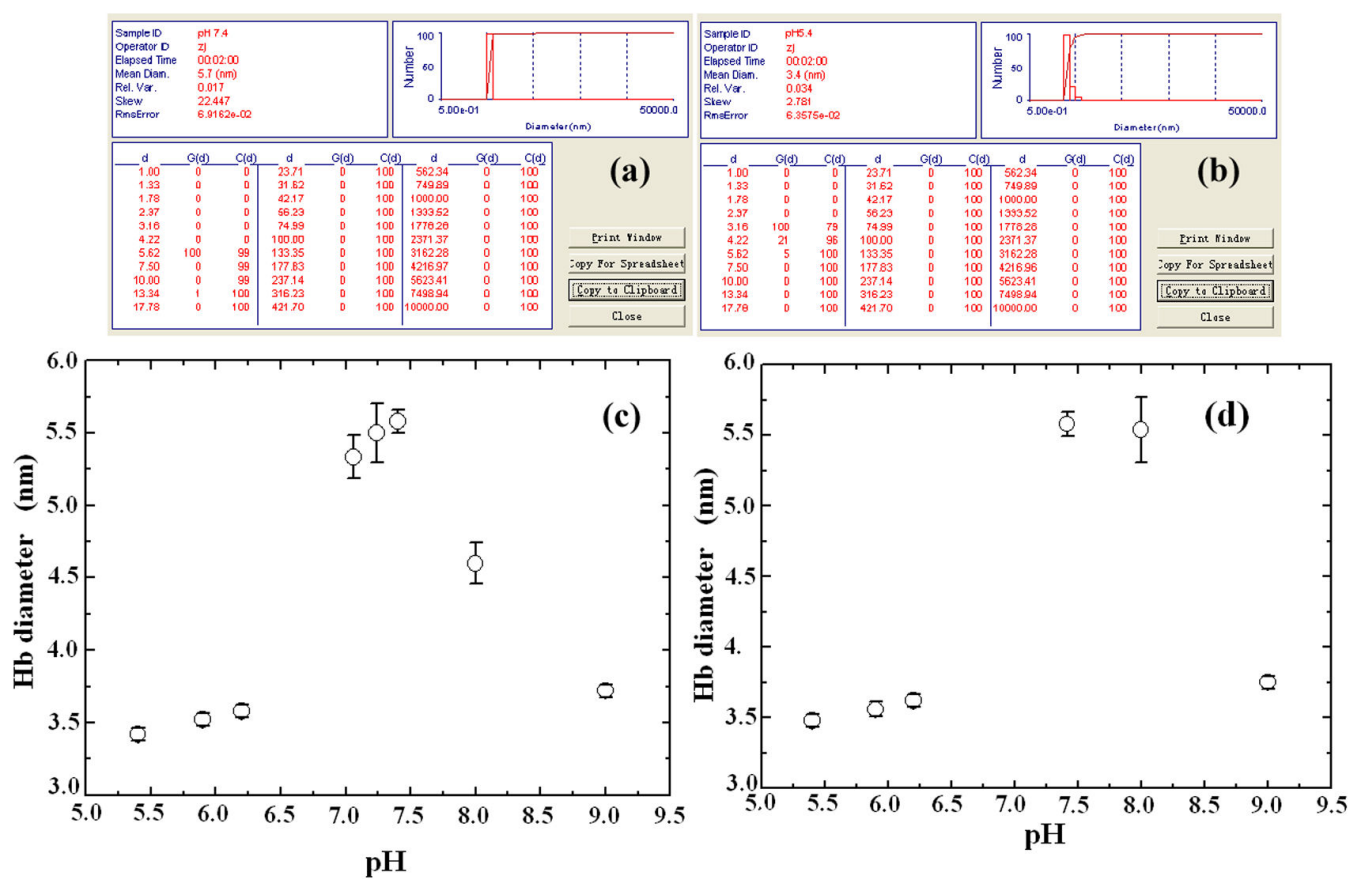
The hydrodynamic diameters and size distributions of human hemoglobin in solutions with different pH levels. (a) The size distribution of Hb at pH 7.4; (b) the size distribution of Hb at pH 5.4; (c) the averaged diameters of human hemoglobin vs. pH level; (d) the averaged hemoglobin diameters in the solutions after their pH levels had been returned to normal condition. The pH values indicated in the horizontal coordinate are the original pH values of the sample solutions.

Figure 1 shows the size distributions of human hemoglobin in pH 7.4 and pH 5.4 solutions respectively. From Figure 1(a) we can see that human hemoglobin in the solution of pH 7.4 was 5.6 nm in size and mono-dispersed. This is consistent with the diameter of a hemoglobin tetramer (5.5 nm) observed by x-ray diffraction[23]. 

Figure 1(b) illustrates the size distribution of hemoglobin in the solution of pH 5.4. We can see that, besides the 5.62 nm tetramers, more particles are either 4.2 nm or 3.16 nm in diameter. According to the formulas[9] about the ratio between the sizes of a tetramer and a dimer:$\frac{R_{T}}{R_{D}}=2^{\frac{1}{3}}$, and between a tetramer and a monomer:$\frac{R_{T}}{R_{M}}=2^{\frac{2}{3}}$, the 4.2 nm and 3.16 nm diameter particles can be distinguished as dimers and monomers respectively. From the table shown in Figure 1(b) about the size distribution of Hb in the pH 5.4 solution, we can see that the majority (^~^80%) of the hemoglobin molecules were monomers, about one-sixth were dimers, and only few were tetramer. 

Figure 1(c) shows how the mean diameter of Hb varies with pH. We can see that the Hb molecules in the physiological range (pH 7.1-7.4) have a mean size of tetramer about 5.3 - 5.6 nm, but become smaller and have a mean size of monomer about 3.4 - 3.7 nm in acidic ( pH 5.4, 5.9, 6.2) and strong alkaline (pH 9.0) conditions. At pH 8.0, the mean size of Hb is 4.6 nm. The mean diameter of Hb at each pH value shown in Figure 1(c) was averaged from the size distribution of hemoglobin in the pH, the size distribution in turn, gives detailed information about the proportions of monomers, dimers and tetramers in each of the hemoglobin solutions. 

Table 1 lists the proportions of monomers, dimers and tetramers in the hemoglobin solutions of different pH values. Each set of the data was averaged from the results of five parallel samples. The proportion of the Hb molecules with a certain size *d* was deduced from the values of G(*d*) (see Figure 1(a) and 1(b)). G(*d*) is the intensity of the light scattered from the particles with size *d* and is proportional to the number of the particles. Therefore, by having all the G(*d*) values of the Hb molecules with different sizes, one can obtain information about the proportion of each kind of the molecules. We can see that in the pH 5.4 solution, most of Hb molecules are monomers. As pH increases, the proportion of monomers decreases, whereas the fraction of dimers increases. In normal pH solution, all the Hb molecules are tetramers. While at pH 8.0, most of Hb molecules are dimers, with some coexisting tetramers but no monomers. When pH value reaches 9.0, once again most of Hb molecules are monomers (about 60%), less than 33% are dimers, and only a few are tetramers (7%). 

**Table 1:** The proportions of monomers, dimers and tetramers in the hemoglobin solutions of different pH levels.

pH	Proportion of Hb with different diameters (%)			Mean diameter(nm)
	3.16nm	4.22nm	5.62nm	
5.4	81.97	15.57	2.46	3.4
	80.65	16.95	2.40	3.4
	82.65	15.70	1.65	3.4
	79.37	16.67	3.96	3.4
	77.52	19.398	3.10	3.4
5.9	67.11	26.17	6.72	3.6
	74.07	21.48	4.45	3.5
	73.53	22.79	3.68	3.5
	68.49	25.35	6.16	3.6
	75.76	20.45	3.79	3.5
6.2	65.36	29.41	5.23	3.6
	68.97	25.52	5.53	3.6
	71.43	23.57	5.00	3.5
	65.36	28.76	5.88	3.6
	70.42	23.94	5.64	3.6
7.4			100	5.6
			100	5.6
			100	5.6
			100	5.6
			100	5.6
8.0	0	81.97	18.03	4.5
	0	82.64	17.36	4.5
	0	83.33	16.67	4.5
	0	79.37	20.63	4.5
	0	76.34	23.66	4.6
9.0	57.47	36.21	6.32	3.7
	59.52	33.33	7.15	3.7
	63.29	30.38	6.33	3.6
	60.97	34.15	4.88	3.6
	57.47	35.06	7.47	3.7

### Gel chromatography

From the Gel chromatography elution profile of a mixture solution of Borvine serum albumin and cytochrome C, it was found that the elution peak of Borvine serum albumin is with a volume of 30.95 ml, while the elution peak of cytochrome C is with a volume of 49.4 ml. By using them as the references, we determined the elution volumes of human hemoglobin in different solutions.

**Figure 2:**
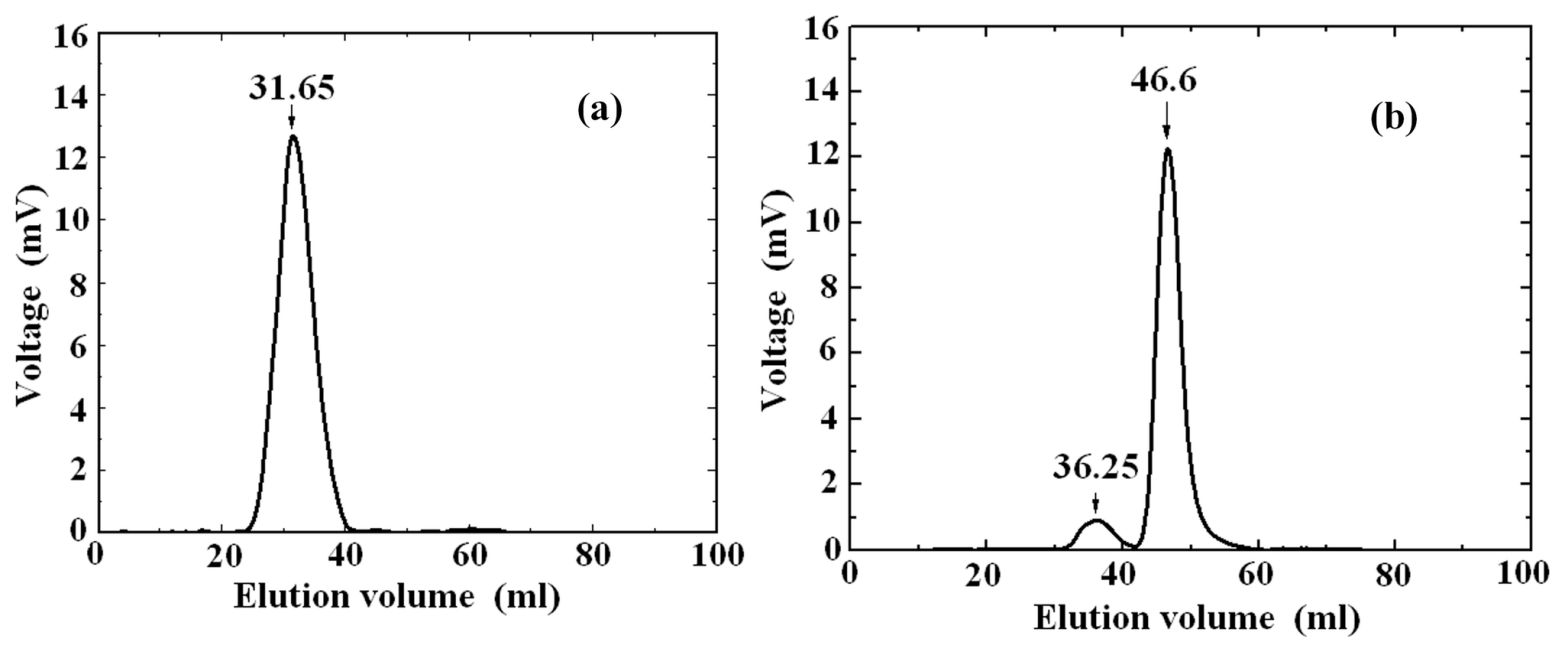
The elution profiles of the hemoglobin solutions (a) at pH 7.4 and (b) at pH 5.4.

Figure 2 (a) displays the elution profile of the hemoglobin solution at pH 7.4. According to the Andrews formula[24], the calculated molecular weight corresponding to the elution volume is about 63KD and should be that of Hb tetramers (64.5KDa). This is consistent with the result of dynamic light scattering measurement.

There are two elution peaks for the solution of pH 5.4 (see Figure 2(b)). The major one has an elution volume of 46.6ml or a molecular weight of 16.39 KD and should be the one of Hb monomers (16 KD). The small one could be the mixture of tetramers (64.5 KD) and dimers (32 KD). The elution profile also suggests that the major molecular structure in the solution is monomer, the same as that implied by the results of dynamic light scattering.

### The reversibility of hemoglobin dissociation

To verify whether or not the dissociated Hb can re-associate to tetramer or dimer, the Hb solutions which had been adjusted to different acidic or alkaline conditions for 72 hours were readjusted to pH 7.4 and incubated for 48 hours (at 4 °C). After that, DLS measurements were performed on the samples at 37 °C to determine their particle size distribution. Figure 1(d) displays the average hemoglobin diameters in the solutions after their pH had been returned to normal condition for 48 hours. The pH values indicated in the horizontal coordinate are the original pH values of the sample solutions. We can see that only the hemoglobin originally in the solution of pH 8.0 can return to a tetramer structure, whereas the hemoglobin molecules in the other solutions which originally were in acidic or other alkaline conditions remained as monomers. 

### Variation of Hb structure in different pH environments depicted by the UV-Visible spectra

**Figure 3:**
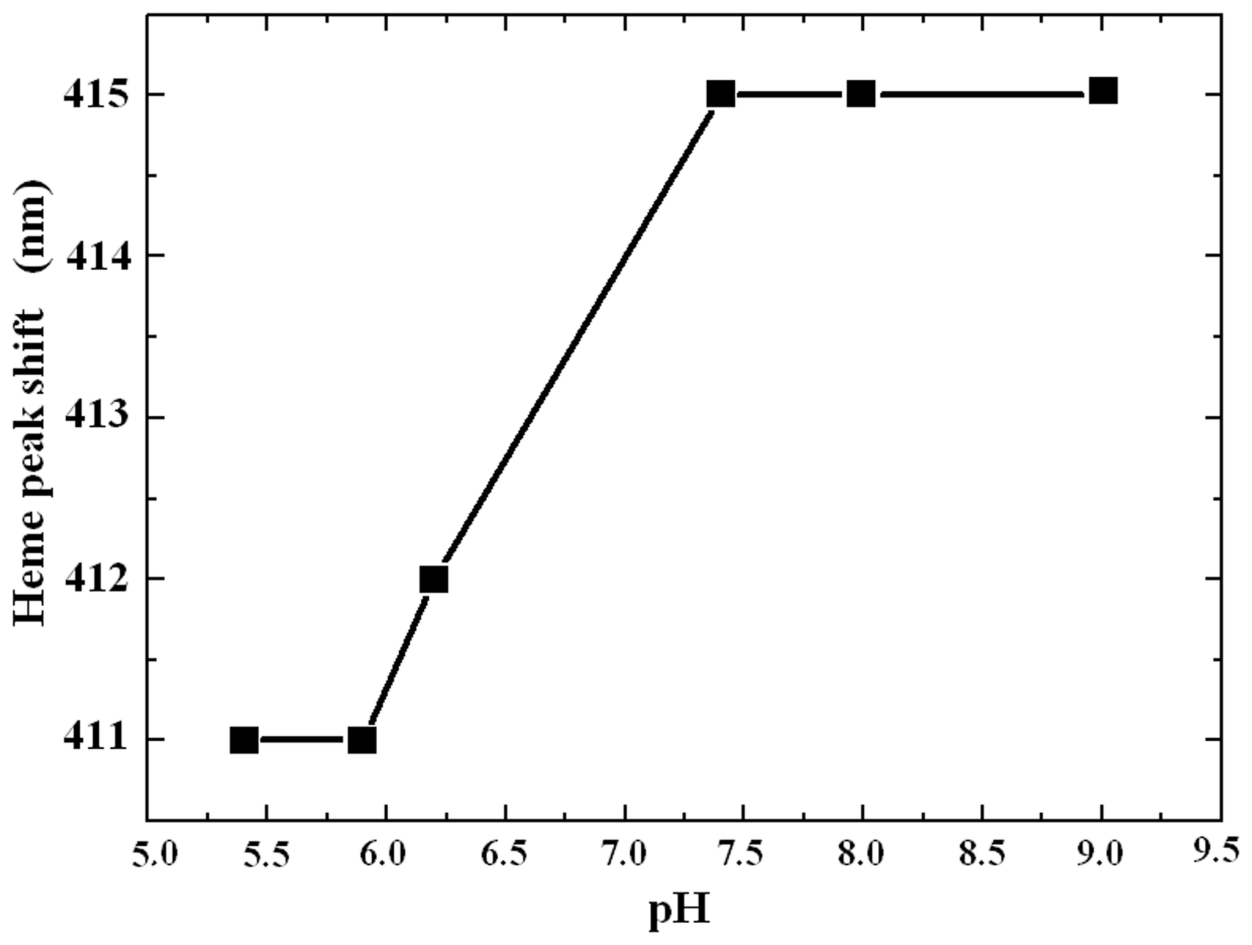
The shift of the heme absorption peak under different pH conditions.

The absorption peak of Hb at 415 nm is the characteristic peak of heme and called the Soret band. Its shift under different pH conditions is displayed in Figure 3. We can see that, when pH value is lower than 7.4, the Soret band has a blue shift, indicating that the combination of heme and the heme socket has changed. However, at higher pH values (8,9), the Soret band doesn’t change.

### Raman spectra of hemoglobin under different pH conditions

**Figure 4:**
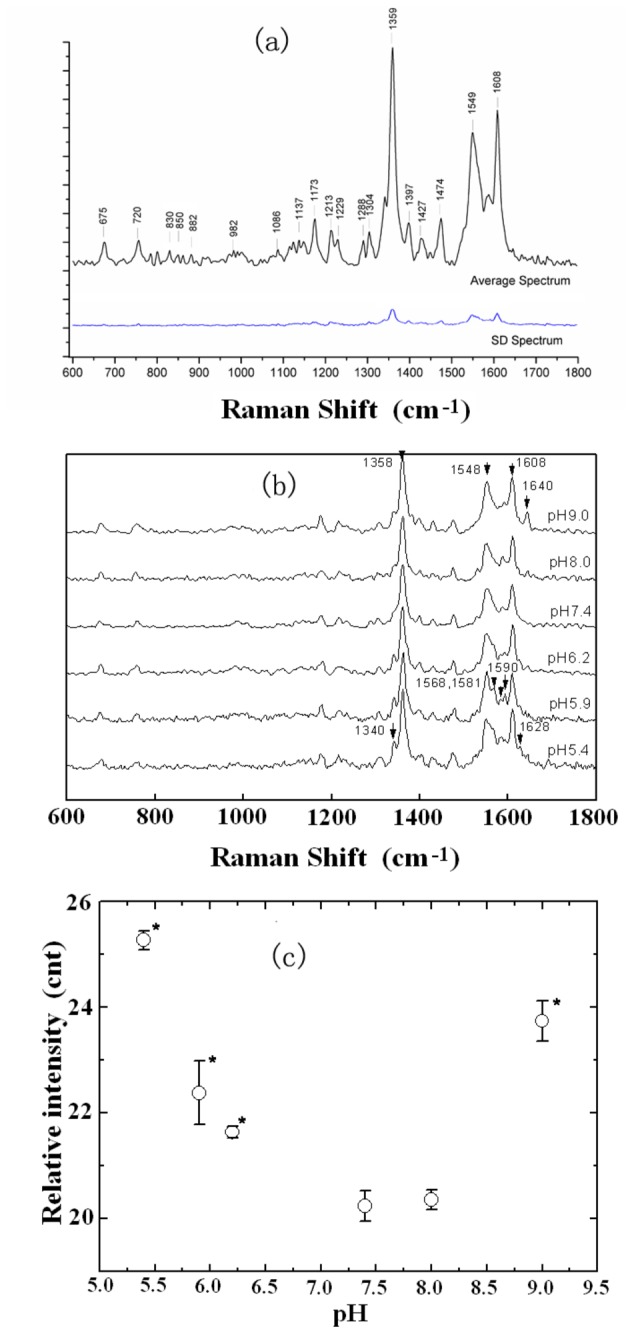
The Raman spectra of hemoglobin. (a) the averaged and standard deviation Raman spectrum calculated from spectra recorded of 25 hemoglobin solutions of pH 7.4; (b) the Raman shifts of human Hb from 600-1800cm^-1^ in various pH conditions and (c) the variation of the peak intensity at 1358 cm^-1^ vs. pH.

Raman spectroscopy measurement can sensitively and accurately detect changes in protein structure, especially the porphyrin moieties within heme and other metalloporphyrin[25]. Figure 4 displays the Raman spectra (from 600-1800 cm^-1^) of hemoglobin in the solutions of different pH values. As mentioned above that, each of the spectra was obtained by averaging the results of at least 25 measurements. No significant spectrum difference was found from different parallel groups, each Raman peak was always in the same position among spectra (1cm^-1^) and the standard deviation in amplitude is relatively small(see Figure 4(a)).

From figure 4(a) we can also see that at pH 7.4, the major Raman peaks of hemoglobin are at 1358, 1548, 1581 and 1608 cm^-1^. These peaks correspond to the characteristic bands of deoxyHb[19,26,27], so it suggests that in normal pH condition hemoglobin is mainly deoxygenated. This is consistent with the results of our previous measurement on the Hb in living human erythrocyte[19]. As described previously, the samples were incubated at 4 °C for 72 hours before Raman spectroscopy measurement. When erythrocytes are cooled down in such a circumstance, the concentration of 2,3 DPG in the cells increases [28,29]. Since 2,3 DPG is a heteroallosteric effector of hemoglobin, it can lower hemoglobin's affinity for oxygen by binding preferentially to deoxyhemoglobin. An increased concentration of DPG in normal red blood cells favors formation of the T, low-affinity state of hemoglobin. Therefore, most of the Hb molecules obtained from the erythrocytes in the condition of 4 °C for 72 hours were in deoxygenated state.

Figure 4(b) shows the Raman spectra of hemoglobin at different pH values. We can see that the peak intensity at 1358cm^-1^ varies with pH. This peak is the ν_4_ band of heme and it is the characteristic band of deoxidized hemoglobin. The increase of the peak intensity with the deviation of pH value from normal (see Figure 4(c)) indicates that in abnormal pH conditions, the heme group becomes exposed, the combination of heme and globin also has changed [20]. Since the ability of hemoglobin to pick up or release oxygen depends on the interaction between oxygen and the iron atom of the heme groups and hemoglobin's quaternary structure, the change in the heme group suggests that the oxygen carrying ability of Hb in abnormal pH is influenced. 

The Raman doublet at 830 and 850 cm^-1^ is due to the Fermi resonance between a ring-breathing vibration and the overtone of an out-of-plane ring bending vibration of Tyr[30], the doublet reflects the status of tyrosine in the globin chain. When the intensity ratio I_850/830_ is less than 1, the tyrosine is “buried”, whereas if the ratio I_850/830_ >1, the tyrosine is “exposed”[31,32].

**Figure 5:**
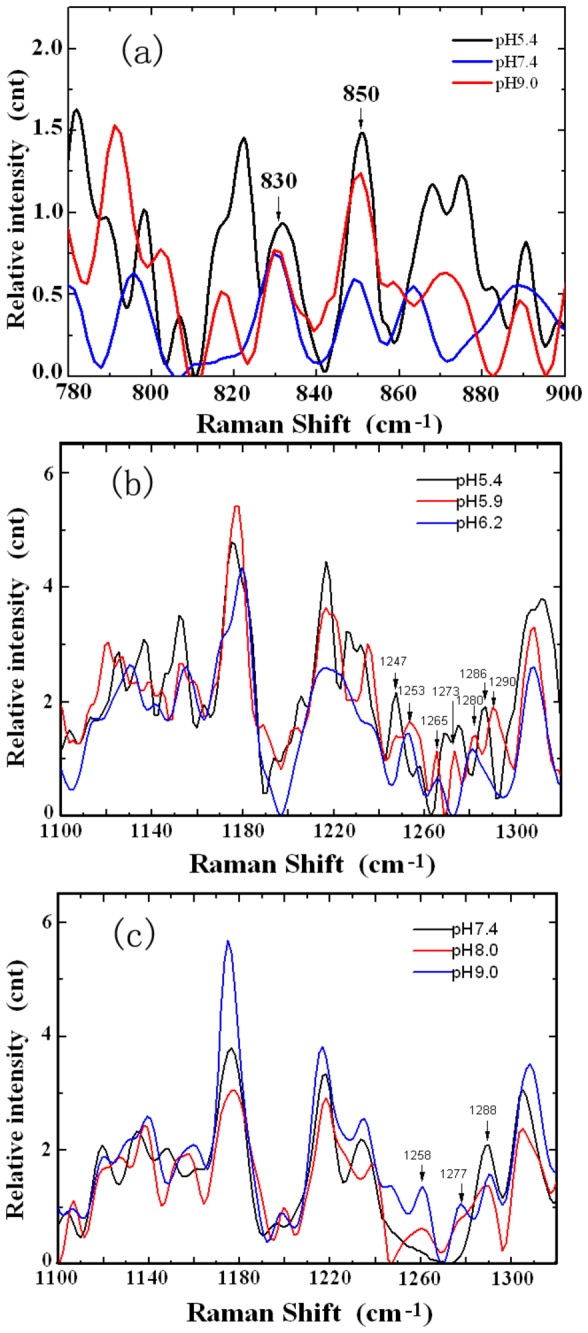
The enlarge images of the Raman spectra of human Hb in various pH conditions averaging from at least 25 spectra measured at each pH value. (a) The Tyr bands at 830cm^-1^ and 850cm^-1^ in solutions of pH 5.4, 7.4 and 9.0 respectively; (b) and (c): the secondary structure transforms in human hemoglobin at different pH levels indicated by enlarged Raman spectra. The 1247, 1253 and 1258 cm^-1^ bands are assigned to the random coil, while the 1265, 1273, 1277, 1280, 1286, 1288 and 1290 cm^-1^ bands are assigned to the α helix of the Amide III mode.

Figure 5(a) gives an enlarged image of the Tyr bands at 830cm^-1^ and 850cm^-1^ in the solutions of pH 5.4, 7.4 and 9.0 respectively. As mentioned above that, each of these spectra and those shown in Figure 5(b) and (c) was obtained by averaging the results of at least 25 measurements at every pH value. Similar to the spectrum and SD shown in Figure 4(a), no significant spectrum difference was found from different parallel groups and the standard deviation in amplitude was relatively small. We can see that in acidic (pH 5.4-6.2) and strong alkaline (pH 9.0) conditions, the intensity of the band at 850 cm^-1^ is greater than that at 830cm^-1^. This indicates that under these conditions, the tyrosine in the globin chains is exposed. Whereas at pH 8.0, the ratio of I_850/830_ is less than 1, which is the same as the case of pH 7.4, suggesting that the tyrosine is buried.

Figure 5(b) and 5(c) are the enlarged images of the bands from 1240 to 1300cm^-1^. The bands in the range of 1240-1260cm^-1^ are assigned to the random coil vibration mode of amide III, and the bands in the 1265-1300cm^-1^ range are assigned to the α helix vibration mode [33,34]. We can see that at normal pH, the characteristic spectra of the α helix vibration mode are evident, suggesting that the secondary structure of hemoglobin mostly exists in α helix vibration mode. As pH is away from neutral, the intensities of the characteristic bands for random coil vibration mode gradually increase, while those for α helix vibration mode decrease. This suggests that in either acidic or strong alkaline condition, the secondary structure of hemoglobin would change from ordered (α helix) to disorder (random coil), so the protein would be damaged. The exception is at pH 8.0; the secondary structure of Hb mainly remains as α helix, indicating that Hb has not undergone any secondary structure change in the circumstance.

Without normal tyrosine and heme group, a Hb molecule cannot bind or release oxygen normally in acid and strong alkaline conditions. Therefore, the Raman spectra obtained in pH 5.4-6.2 and pH 9.0 (see Figure 4(a)) are quite complex. They exhibit not only the characteristic peaks of deoxyHb (1358, 1548, 1581, 1608cm^-1^), but also the characteristic peaks of oxyHb (1568, 1590 and 1640cm^-1^) and even some of metHb (1340 and 1628cm^-1^). 

### Mechanism of Hb dissociation from tetramer to dimer

Human hemoglobin includes four subunits, two α globin chains and two β globin chains, which held together by non-covalent bonds in a globular tetramer configuration. It was believed that the stability of a hemoglobin tetramer is relevant to electrostatic interactions[35], which also influences the dissociation of hemoglobin from tetramer into dimer. Taking the Hb dissociation process in pH 8.0 as an example, the mean diameter of a Hb particle is 4.52 ± 0.04 nm and the hemoglobin concentration is 100 μM (heme). Hence the electrostatic free energy calculated with the model derived by Tanford, etc.[9,36] is: ΔG_el_ = −3.31 ± 0.20 RT. This indicates that under the condition the electrostatic free energy of a dimer is lower than that of a tetramer, so tetramer favors to dissociate into dimer. The calculated tetramer-dimer equilibrium constant K_4_ is 1722.43 ± 303.42 μM (heme), also suggesting that hemoglobin in this circumstance is prone to exist in the form of dimmer.

Theoretically, tetramer α_2_β_2_ can dissociate into dimer in three pathways: symmetric dissociation (α_2_β_2_→2αβ), asymmetric dissociation (α_2_β_2_→α_2_+β_2_) and nonspecific dissociation (2α_2_β_2_→α_2_+β_2_+2αβ). But the actual hemoglobin dissociation is mainly subjected to a highly symmetric mechanism (α_2_β_2_→2αβ). For the α-subunit and β-subunit are mainly associated by non-polar residues to form a stable interface. The α- and β-subunits that interacting along the α_1_β_1_ (α_2_β_2_) packing interface form tight contacts and involve about 34 residues, whereas the α_1_β_2_ (α_2_β_1_) sliding interface involves only about 19 residues and is characterized by weaker contacts. Consequently, dissociation often happens at α_1_β_2_ or α_2_β_1_ sliding interface. So the hemoglobin dissociation from tetramer to dimer can only result in the formation of αβ dimer but not α_1_α_2_ or β_1_β_2_ dimer. 

### Mechanism of Hb dissociation from tetramer to monomer

α_1_β_1_ (or α_2_β_2_) is a stable interface of subunit αβ- and usually difficult to further dissociate, its dissociation to monomer at acidic and strong alkaline conditions should be of a mechanism differing from that of tetramer → dimer. According to our experimental results of Raman scattering spectroscopy, the secondary and tertiary structures of hemoglobin had changed in the process of the dissociation. The mechanism of the dissociation is proposed as follows. 

As shown in Figure 5(a), the hydrophobic amino acid has changed from burial to exposure under acidic and strong alkaline conditions. So the hemoglobin dimer cannot keep its stability but dissociates into monomer. 

Another secondary structure change of the Hb under pH 5.4-6.2 and pH 9.0 conditions is that the α-helix has been replaced by random coil. The α-helix structure is the basis for the formation of a tertiary structure with particular conformation for a stable Hb, the loss of α-helical structure for hemoglobin strongly suggests local perturbations in globin, such as the interactions among the subunits are significantly weakened and some weak salt-bridge linked among each of them are even broken. So that dimer is facilitated to dissociate into monomer. 

The third change is in the structure of heme pocket as shown in Figure 3. The Raman spectroscopy of the pyrrole ring shown in Figure 4(a) also suggests that there is a displacement of heme in the heme pocket which would expose it to the solution environment. This was further proved by the increase of the intensity of the characteristic spectral band of heme at 1358cm^-1^ shown in Figure 4(b). Since the more “exposed” heme would absorb more light to increase the intensities of the characteristic bands[20], heme should be exposed in the circumstance. The exposure to the solvent of the hydrophobic surface in the inter-subunit interfaces is one of the most important features of protein unfolding, some other proteins were also found to be dissociated from tetramers to non-native monomers due to the effect [37,38].

 Therefore, these changes in the secondary and tertiary structures would disrupt the formation of the secondary bond that maintains the stability of the α_1_β_1_ interface, thus resulting in the dissociation from dimer to monomer. One may argue that besides the dissociation from dimer to monomer, it may not get rid of the possibility that hemoglobin tetramer directly dissociates into monomer. However, based on our experimental evidences, this possibility should be quite rare. Since the dimer structure was always found in all the solutions with various proportions under different pH conditions. Its proportion increases as pH value approaching to the physiological condition. If hemoglobin dissociates directly from tetramer to monomer, there should be only monomer existing in acidic and strong alkaline conditions but without dimer. Therefore, tetramer Hb should dissociate first into dimer, then to monomer.

### Mechanism of the reversibility of hemoglobin dissociations

As shown in Figure 1(d), the dissociation of hemoglobin from tetramer to dimer is reversible, whereas that from dimmer to monomer is irreversible. The reversibility of Hb dissociation from tetramer to dimmer can be explained by using Tanford’s electrostatic free energy model [9,36]. Based on the model, the electrostatic free energy difference between dimer and tetramer at pH 7.4 is ΔG_el_ = 0.75 ± 0.97 RT, so hemoglobin favors to exit in the form of tetramer structure at pH 7.4. The equilibrium constant K = 25.65 ± 22.48 μM (heme) in the condition also suggests that the α_2_β_2_↔ 2αβ processes can be on reversible direction. Both Raman and UV-visible absorption spectroscopy measurements showed that the re-associated Hb has the same molecular structures and oxygen carrying function as that of normal Hb at pH 7.4, indicating that the re-associated hemoglobin is active again as it returns to the tetramer form.

In contrast, the dissociation from dimer to monomer is caused by structural changes of the hemoglobin subunits. These changes in subunit structure are difficult to recover, so when the pH environment returns to 7.4, the hemoglobin monomer cannot re-associate to dimer or tetramer. 

## Conclusions

In summary, we have conducted a multi-technique systematic study on the effect of pH on the structure and function of human hemoglobin, and demonstrated with direct and convincing evidences that when the environmental pH is away from normal physiological value, the tetramer hemoglobin would easily dissociate into dimer by having electrostatic free energy advantage. The tetramer → dimer dissociation is a α_2_β_2_ → 2αβ process and it is reversible if the environmental pH returns to neutral value. When pH becomes more acidic and alkaline, such as in pH 5.4-6.5 and pH 9.0, dimer Hb will further dissociate into monomer. The dissociation is accompanied with series changes of protein structure, so that the secondary bond is unable to form between the subunits to maintain a stable state of dimer, thus causing the dissociation of dimer and inducing the ferrous iron transform to ferrate iron by peroxidization. Since the dissociation process involves structure changes, even if the environmental pH returns to 7.4, it is not reversible. The dissociated Hb is not able to adequately carry and release oxygen to the tissues in circulation. Therefore, pH dependent Hb dissociation should be avoided in patients and preserved blood.
